# miR-96-5p is involved in alcohol-induced apoptosis in PC12 cells via negatively regulating TAp73

**DOI:** 10.1371/journal.pone.0282488

**Published:** 2023-04-26

**Authors:** Bin Yang, Qi Wang, Yanzhong Li, Lin Li, YanJie Zhang, Mohammad Farris Iman Leong Bin Abdullah, Wei Hao, Duan Li, Ruiling Zhang

**Affiliations:** 1 The Second Affiliated Hospital of Xinxiang Medical University, Henan Mental Hospital, Xinxiang, Henan, China; 2 Henan Key Lab of Biological Psychiatry, International Joint Research Laboratory for Psychiatry and Neuroscience of Henan, Xinxiang, Henan, China; 3 Department of Community Health, Advanced Medical and Dental Institute, Universiti Sains Malaysia, Kepala Batas, Pulau Pinang, Malaysia; 4 Department of Microbiology, School of Basic Medical Sciences, Xinxiang Medical University, Xinxiang, Henan, China; National Institutes of Health, UNITED STATES

## Abstract

**Objective:**

The present study opted for the adrenal phaeochromocytoma (PC12) cell line to frame a neuronal injury model induced by alcohol exposure in vitro, aiming to probe whether TAp73 and miR-96-5p are involved in the neuronal injury process induced by alcohol and elucidate the regulatory relationship between miR-96-5p and TAp73.

**Methods:**

Immunofluorescence staining was used to observe the structural features of PC12 cells after culturing in medium with nerve growth factor (NGF). After different doses and different durations of alcohol treatment, CCK-8 assay was performed to detect the viability of PC12 cells, flow cytometry assay was carried out to detect the apoptosis rate of PC12 cells, dual-luciferase reporter assay was used to definitude the regulatory relationship between miR-96-5p and Tp73, and western blot was used to detect the protein expression of TAp73.

**Results:**

The result of immunofluorescence staining demonstrated that PC12 cells abundantly expressed Map2, CCK-8 assay illustrated alcohol exposure significantly downregulated the cell viability of PC12 cells, Treatment with miR-96-5p inhibitor induced apoptosis and upregulated the expression of TAp73 in PC12 cells. Contrastingly, miR-96-5p mimic reversed the above effects and downregulation of TAp73 inhibited the apoptosis of PC12 cells.

**Conclusion:**

The present study demonstrated that miR-96-5p participates in alcohol-induced apoptosis in PC12 cells via negatively regulating TAp73.

## 1. Introduction

Alcohol abuse has become a major medical and social problem worldwide. The 2018 Global Drinking and Health Report released by the World Health Organization indicated that the number of people who consumed alcohol in the world was 2.3 billion in 2016. Alcohol use exerts negative impact as it is related to risk of developing 230 diseases [1]. The global burden of disease study released by the Lancet revealed that alcohol abuse had climbed to third place among all risk factors which predisposed to occurence of diseases from 1990 to 2010 [2].

Alcohol exposure has led to a series of social problems, such as health issues and family conflicts [2]. Current studies have shown that alcohol exposure inflicts varying degrees of injury to different tissues and organs [3]. For example, alcohol can cross the blood–brain barrier and cause oxidative stress in the central nervous system, which can lead to neuronal apoptosis [4]. However, the specific mechanisms of alcohol-induced neuronal apoptosis remain to be further studied. Therefore, it is of utmost importance to explore the molecular mechanism underlying the effect of alcohol exposure to neuronal injury to uncover new diagnostic and treatment methods.

As one of the p53 family, p73 participates in many aspects of cell life activities [5]. p73 is divided into two isoforms: TAp73, which includes the full-length N-terminal sequence, DNp73, which lacks the N-terminal trans-activation domain [6]. Under normal physiological conditions, TAp73 and DNp73 maintain a certain ratio to maintain the body’s homeostasis. However, in some pathophysiological conditions, an imbalance between the ratios of TAp73 and DNp73 can often be observed. Increased proportion of DNp73 can counteract the cell apoptotic effect of TAp73, while increased proportion of TAp73 can induce apoptosis by activating a series of pro-apoptotic factors in cells [7]. However, it remains indistinct whether TAp73 partakes the process of alcohol exposure-induced apoptosis and the detailed mechanism needs to be further explored.

As a type of naturally generated short-chain noncoding RNA, mature microRNAs (miRNAs) have been found to participate in various physiological and pathological processes [8]. miRNAs are abundantly expressed in the central nervous system, are sensitive to alcohol, and are involved in the development of alcoholism [9]. Our previous study screened the differential expression of miRNAs in the hippocampus of alcohol-dependent rats by high-throughput sequencing, the results illustrated a conspicuous reduced trend of the expression of miR-96-5p, Fold Change = -2.49, which prompts that miR-96-5p may partake in the process of alcohol exposure-induced injury. However, the detailed mechanism needs to be further explored.

Therefore, in this research, we used PC12 cells to establish a neuronal injury model induced by alcohol exposure to probe the potential mechanism of TAp73 and miR-96-5p involvement in alcohol exposure-induced cell injury.

## 2. Materials and methods

### 2.1 Cell culture and treatment

The PC12 cell line used in this study was acquired from the American Type Culture Collection (VA, USA). All procedures were approved by the Animal Care and Use Committee of Xinxiang Medical University. PC12 cells were nourished in DMEM (Gibco, NY, USA) supplemented with 5% fetal bovine serum (PAN Biotech, Aidenbach, Germany), 5% horse serum (Gibco, NY, USA), 100 U/mL penicillin/streptomycin (BI, Kibbutz Beit-Haemek, Israel), and 110 μg/mL sodium pyruvate solution (BI, Kibbutz Beit-Haemek, Israel). PC12 cells were nourished at 37°C in a humidified atmosphere of 5% CO_2_. Before complying all experiments in this research, PC12 cells were nourished with 50 ng/mL rat NGF (Gibco, NY, USA) for 48 h to induce the formation of neuron-like properties. Then after seeding in suitable petri dishes, PC12 cells were used for follow-up experiments.

PC12 cells were planted in 24-well culture plates and maintained for 24 h until they reached 60% confluence. The cells were transfected with 50 nM or 100 nM ribo TRACER^™^ (RiboBio, Guangzhou, China) for 24 or 48 hours. Finally, images were obtained using a confocal laser scanning microscope (Leica, Wetzlar, Germany).

Mimics, inhibitors, mimic negative control (mimic NC), inhibitor negative control (inhibitor NC), siRNA TAp73 and siRNA negative control (siRNA NC) were purchased from RiboBio (Guangzhou, China). Cells were first cultured to approximately 50–60% confluence, and then cells were transfected using Lipofectamine 2000 (RiboBio, Guangzhou, China).

### 2.2 Immunofluorescence

Immunofluorescence was performed as previously described [10]. PC12 cells were first fixed with 4% paraformaldehyde at room temperature for 15 min, then nourished with 0.25% Triton X-100 for 12 min on ice and followed by incubation in goat serum for 60 min. Next step, PC12 cells were nourished with antibodies specific for mouse Map2 (GeneTex, CA, USA) overnight at 4°C and then incubated at 37°C with Alexa Fluor^™^ 488 Donkey anti-mouse IgG (H + L) (Invitrogen, CA, USA). Eventually, PC12 cells were counterstained with DAPI and graphics were obtained by a confocal laser scanning microscope (Leica, Ernst-Leitz-Strasse, Wetzlar, Germany).

### 2.3 Dual-luciferase report assay

PC12 cells was nourished in 6-well plates for dual-luciferase report assay. After 24 h, the confluence of PC12 cells reached 60%. PC12 cells were cotransfected with miR-96-5p mimic together with Psi-Tp73-3′-UTR wt or Psi-Tp73-3′-UTR mutant reporter plasmids using Lipofectamine 2000. A dual-luciferase reporter assay system (Promega, WIS, USA) was used to detect firefly and renilla luciferase activities.

### 2.4 CCK-8 assay

The CCK-8 assay was fulfilled as formerly depicted [10]. The cell activity of PC12 cells was investigated using a CCK-8 assay (MCE, NJ, USA). PC12 cells were planted in 96 plates (4 × 10^4^ cells/well) and exposed to 50 mM, 100 mM, 150 mM, and 200 mM alcohol for 6 h or 50 mM alcohol for 3 h, 6 h, 9 h, and 12 h. 10 μL of CCK-8 solution was supplemented into each well, then the cells were nourished for 2 h at 37°C. Finally, the cell activity of PC12 cells was detected at 450 nm using Multiskan Go (Waltham, MA, USA). Cell viability was determined and computed using the following formula: [OD (experimental group)-OD (blank)] / [OD (control group) -OD (blank)].

### 2.5 Flow cytometry assay

Flow cytometry was fulfilled as formerly depicted [10]. According to the manufacturer’s instructions, after various preset processing, PC12 cells were slightly gathered and cleaned with PBS twice, then nourished with Annexin V-FITC and PI (BD Biosciences, CA, USA) at room temperature and avoided light for 15 min. Final results were immediately detected by keeping an account of and analyzing 10,000 cells with a flow cytometer (BECKMAN, CA, USA).

Live cells are not labeled with propidium iodide and annexin V-FITC. Early apoptotic cells and necrotic cells are respectively stained by annexin V-FITC and propidium iodide. Late apoptotic cells are marked by both propidium iodide and annexin V-FITC.

### 2.6 Western blot assay

Western blot assay was fulfilled as formerly depicted [11]. Total protein (50 μg of protein/lane) was put into SDS–PAGE gels, then incubated by electrophoresis and delivered to PVDF membranes. Subsequently, the target areas of the membranes were incubated with rabbit antibodies for anti-TAp73 (1:1000, Abcam, MA, USA) and anti-β-actin (1:2000, Beyotime, Shanghai, China) overnight at 4°C. Then, the target areas of the membranes were hatched with goat anti-rabbit IgG (H+L) secondary antibody (Invitrogen, CA, USA) and detected by X-ray film with an enhanced chemiluminescence system. ImageJ 1.48 (National Institutes of Health, Maryland, USA) was applied to investigate the intensity of the bands.

### 2.7 Statistical analysis

In this study, SPSS 22.0 was employed for statistical analysis. The results of Kolmogorov-Smirnov test indicated that all data obey normal distribution in all groups (*P* > 0.1). All data were presented as the mean ± SEM. This study employed the multiple comparison one-way analysis of variance and post hoc LSD t-test to analyze the data. Statistical significance was set at *P* < 0.05, which is indicated by *, while *P* < 0.01 is indicated by **.

## 3. Results

### 3.1 Neuron-like properties of differentiated PC12 cells

**Fig 1:**
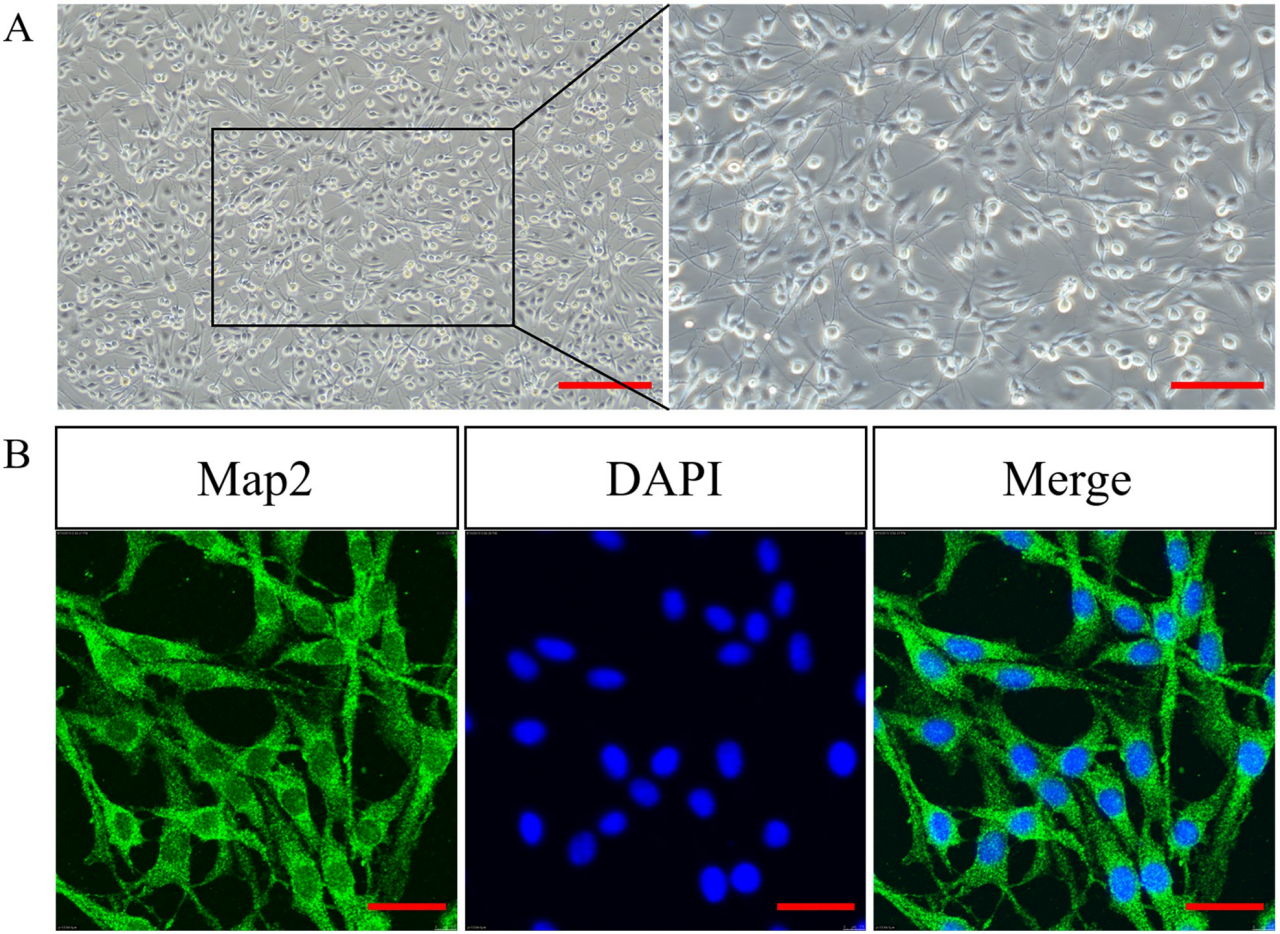
(A) The neuron-like characteristics of PC12 cells after NGF treatment. Left: bars = 200 μm, right: bars = 50 μm. (B) The expression of Map2 in PC12 cells. Bars = 25 μm. Map2: microtubule-associated protein 2.

The morphological characteristics of differentiated PC12 cells were polygonal or long fusiform with abundant protrusions connected between cells (Fig 1A). Differentiated PC12 cells abundantly expressed Map2 (Fig 1B).

### 3.2 Cell viability of PC12 cells

**Fig 2:**
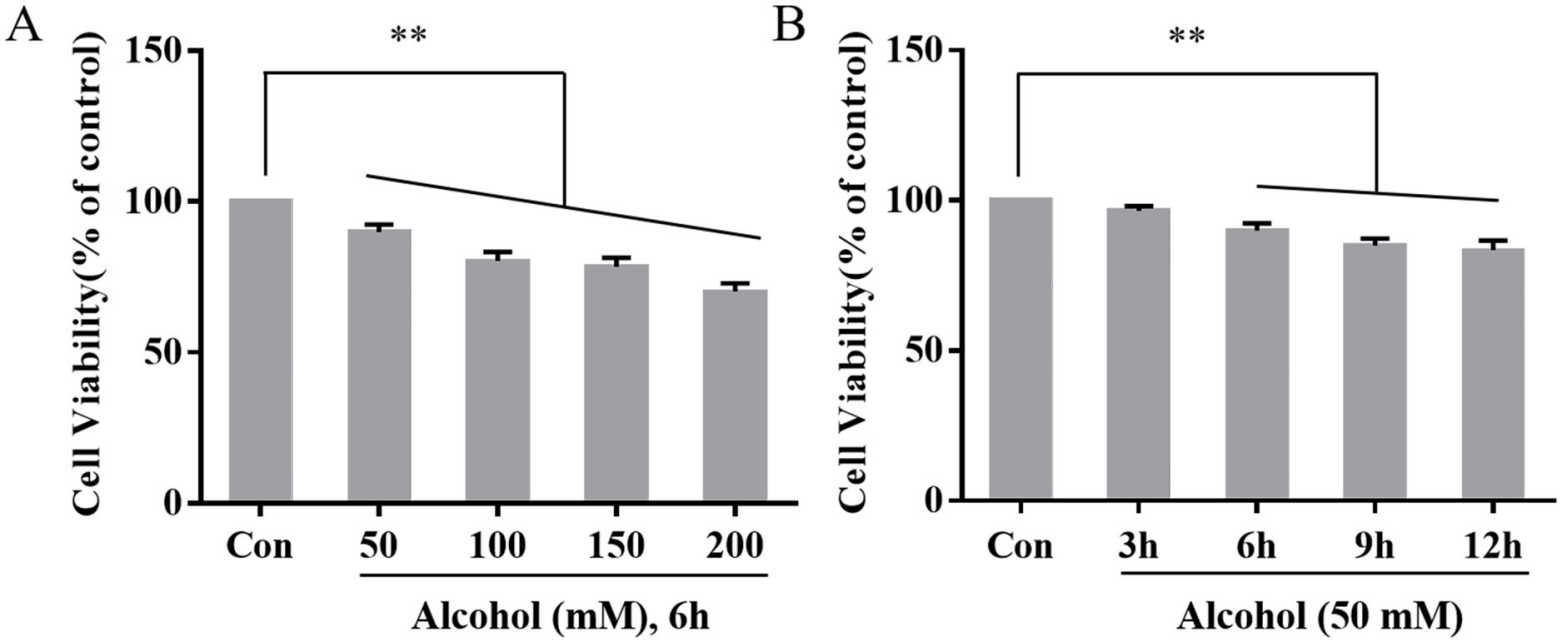
The effect of different doses and different durations of alcohol treatment on the viability of PC12 cells (n = 6). Data information: bars are the mean ± SEM, **P < 0.01 (highly statistically significant).

Significantly different from the control group (100.0 ± 0.0%), the viability of PC12 cells appeared a downward trend with increasing alcohol concentration (50 mM (89.98 ± 2.42%, *P* < 0.01), 100 mM (80.29 ± 3.08%, *P* < 0.01), 150 mM (78.40 ± 2.90%, *P* < 0.01) and 200 mM (70.19 ± 2.77%, *P* < 0.01)) (Fig 2A).

Significantly different from the control group, the viability of PC12 cells showed no difference after processed by 50 mM alcohol for 3 h (96.53 ± 1.58%, *P* > 0.05) but appeared a downward trend at 6 h (89.98 ± 2.42%, *P* < 0.01), 9 h (85.06 ± 2.29%, *P* < 0.01), and 12 h (83.39 ± 3.32%, *P* < 0.01). Based on these results, 50 mM alcohol was used to treat PC12 cells for 6 h in subsequent experiments (Fig 2B).

### 3.3 Regulatory relationship between miR-96-5p and Tp73

**Fig 3:**
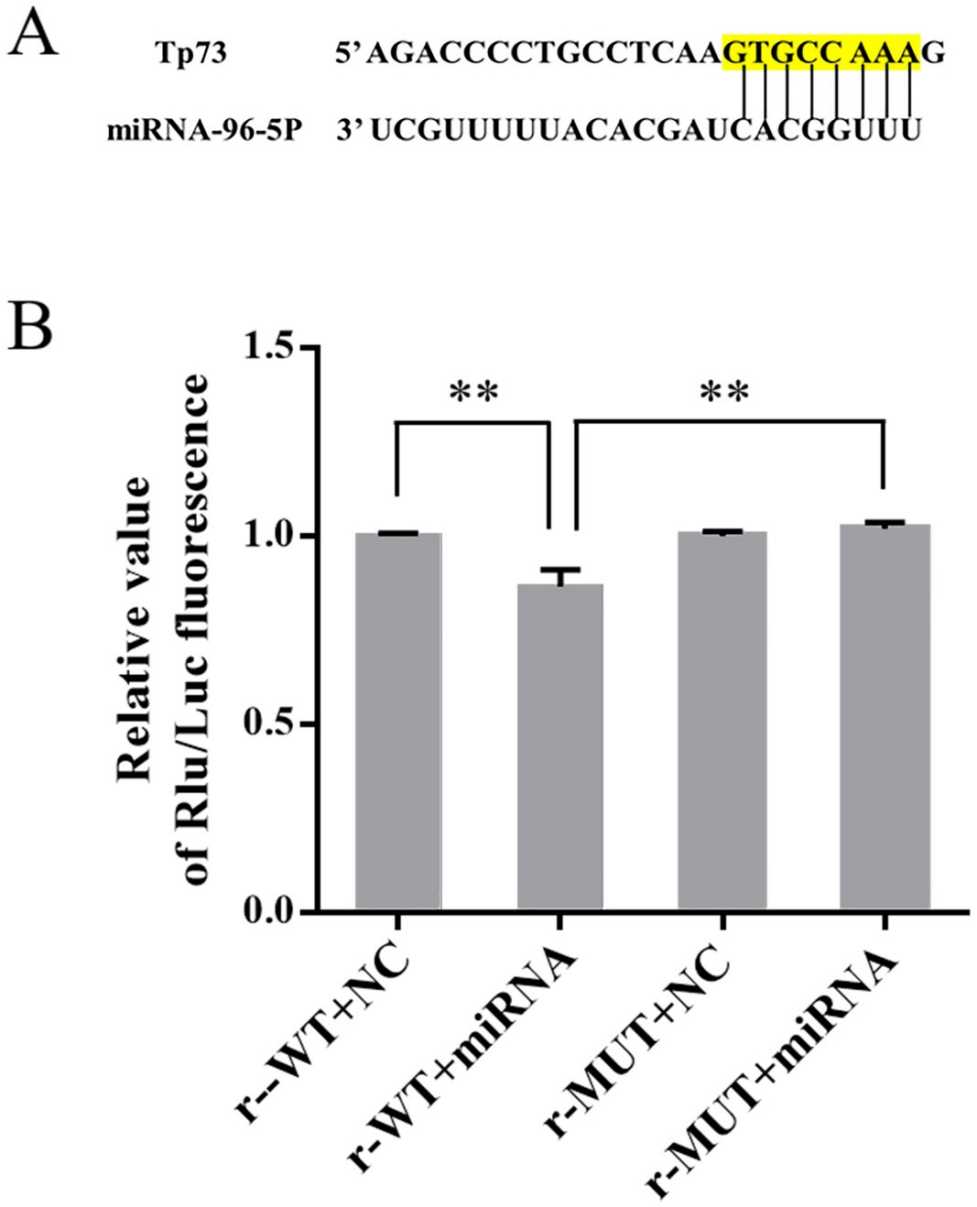
(A) The complementary base sequence for miR-96-5p and Tp73. (B) Luciferase reporter plasmids that contained the Tp73 3’-UTR with wild-type or mutant miR-96-5p binding sites were cotransfected with equal quantities of either the miR-96-5p mimic or mimic NC (n = 4). Data information: bars are the mean ± SEM, **P < 0.01 (highly statistically significant).

The nucleic acid sequences of miR-96-5p and Tp73 mRNA illustrated that the 3’-UTR regions of Tp73 gene included a matching site with the sequence of miR-96-5p (Fig 3A).

Compared to the r-WT + NC group (1.00 ± 0.01), the fluorescence value was significantly downregulated in the r-WT + miRNA group (0.87 ± 0.02, *P* < 0.01); after mutation with the expected target site of Tp73, the fluorescence value significantly increased in the r-MUT + miRNA group (1.02 ± 0.02, *P* < 0.01) (Fig 3B).

### 3.4 Determination of transfection time

**Fig 4:**
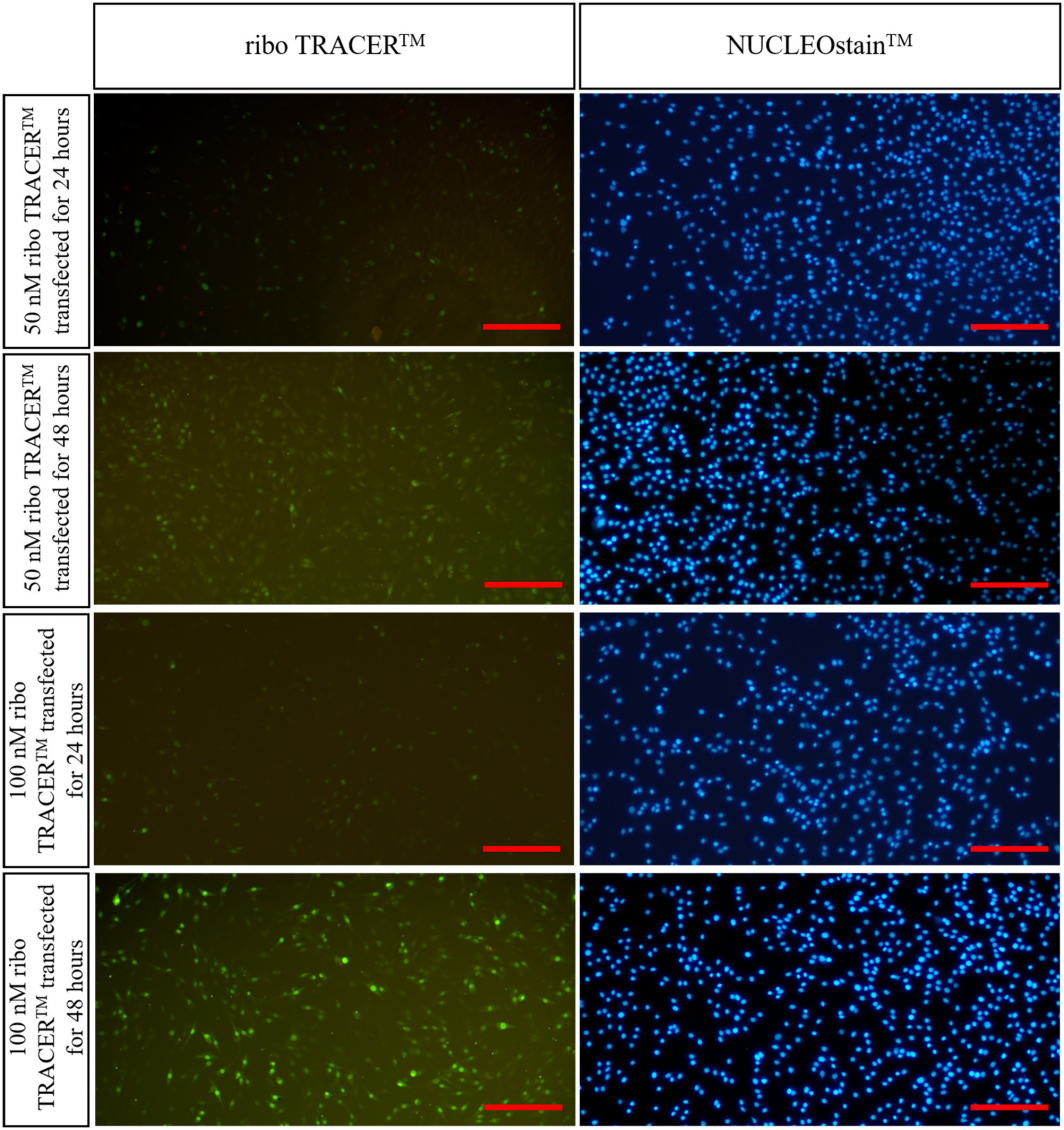
The transfection efficiency of PC12 cells incubated with 50 nM or 100 nM ribo TRACERTM for 24 h or 48 h (n = 3). bars = 200 μm.

Compared to the transfection efficiency of PC12 cells incubated with 50 nM or 100 nM ribo TRACER^™^ for 24 h, the transfection efficiency of PC12 cells incubated with 50 nM or 100 nM ribo TRACER^™^ for 48 h was significantly increased (Fig 4). Based on this result, the duration of PC12 cell incubation with mimic and inhibitor was set at 48 h (Fig 4).

### 3.5 The effect of the miR-96-5p mimic on expression of TAp73 and the apoptosis rate of PC12 cells

**Fig 5:**
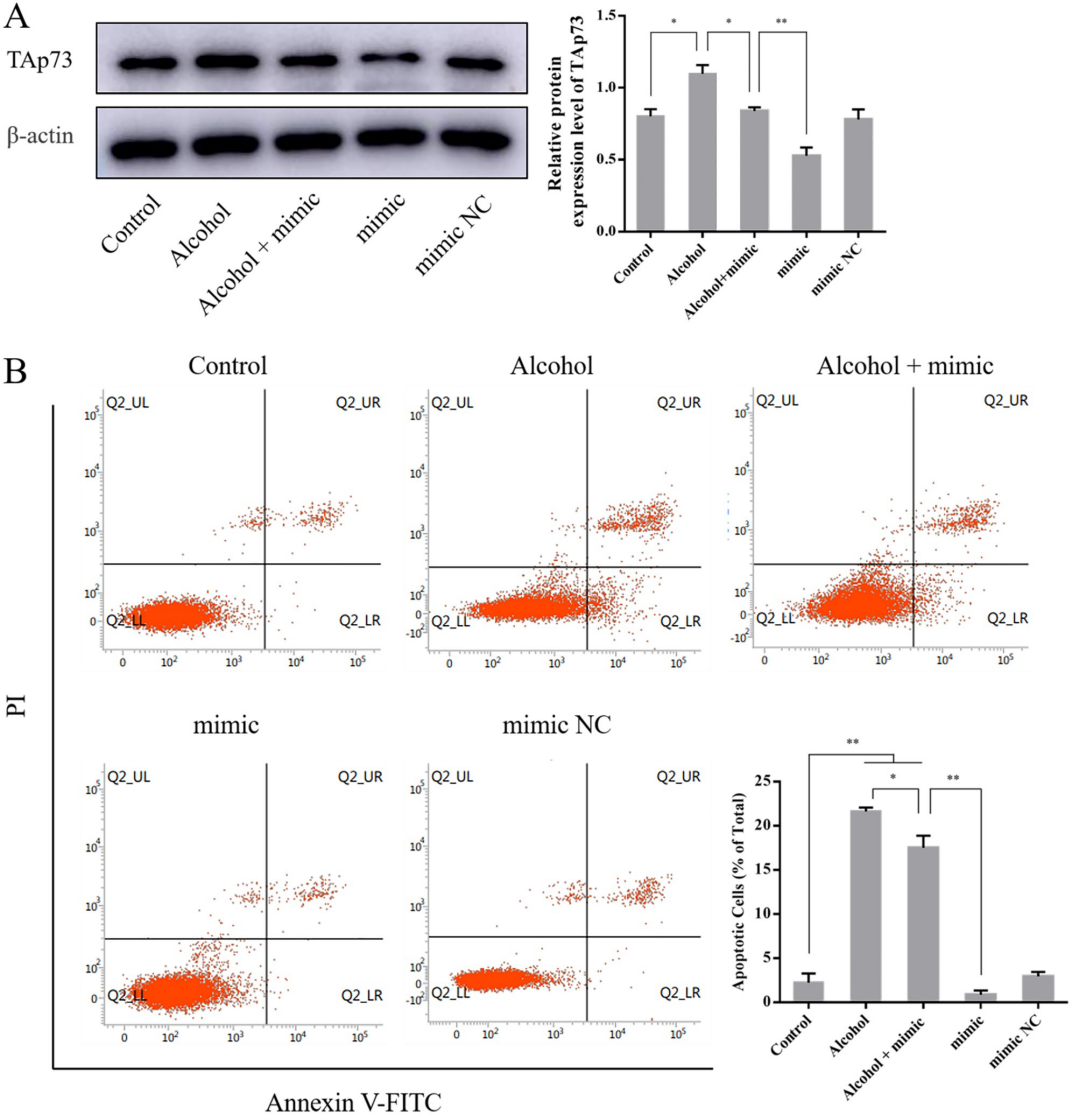
(A) The effect of the miR-96-5p mimic on expression of TAp73 in PC12 cells (n = 3). (B) The effect of the miR-96-5p mimic on the apoptosis rate of PC12 cells (n = 3). Data information: bars are the mean ± SEM, *P < 0.05 (significant difference) and **P < 0.01 (highly statistically significant).

Compared to the control group (0.80 ± 0.05), TAp73 expression was significantly upregulated in the alcohol group (1.10 ± 0.06, *P* < 0.05). Compared to the alcohol group, TAp73 expression was significantly downregulated in the alcohol + mimic group (0.84 ± 0.03, *P* < 0.05), and compared to the mimic group, TAp73 expression was significantly upregulated in the alcohol + mimic group (0.53 ± 0.06, *P* < 0.01) (Fig 5A).

Compared to the control group (2.25 ± 1.03%), the apoptotic status of PC12 cells stayed at a low level in the mimic NC group (2.96 ± 0.49%, *P* > 0.05) but were significantly increased in the alcohol group (21.64 ± 0.43, *P* < 0.01) and alcohol + mimic group (17.49 ± 1.39, *P* < 0.01). Compared to the alcohol group, the apoptosis rate of PC12 cells was significantly decreased in the alcohol + mimic group (*P* < 0.05). Compared to the mimic group, the apoptosis rate of PC12 cells was significantly increased in the alcohol + mimic group (0.88 ± 0.47, *P* < 0.01) (Fig 5B).

### 3.6 The effect of the miR-96-5p inhibitor on expression of TAp73 and the apoptosis rate of PC12 cells

**Fig 6:**
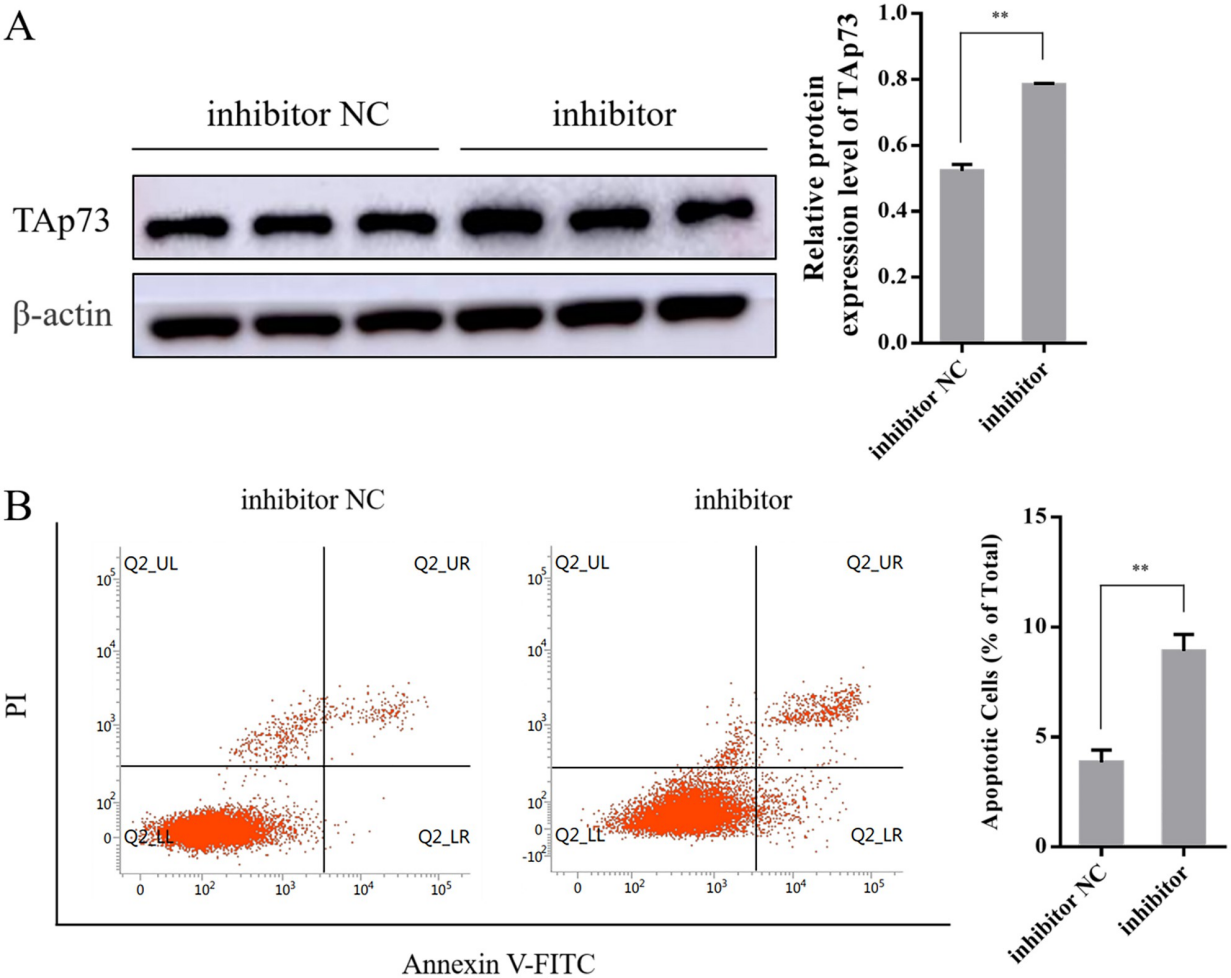
(A) The effect of the miR-96-5p inhibitor on the expression of TAp73 in PC12 cells (n = 3). (B) The effect of the miR-96-5p inhibitor on the apoptosis rate of PC12 cells (n = 3). Data information: bars are the mean ± SEM, **P < 0.01 (highly statistically significant).

Compared to the inhibitor NC group (0.52 ± 0.02), expression level of TAp73 was significantly upregulated in the miR-96-5p inhibitor group (0.79 ± 0.01, *P* < 0.01) (Fig 6A).

Compared to the inhibitor NC group (3.86 ± 0.55), the apoptotic status of PC12 cells was obviously increased in the miR-96-5p inhibitor group (8.91 ± 0.76, *P* < 0.01) (Fig 6B).

### 3.7 The effect of TAp73 siRNA on the expression of TAp73 and the apoptosis rate of PC12 cells

**Fig 7:**
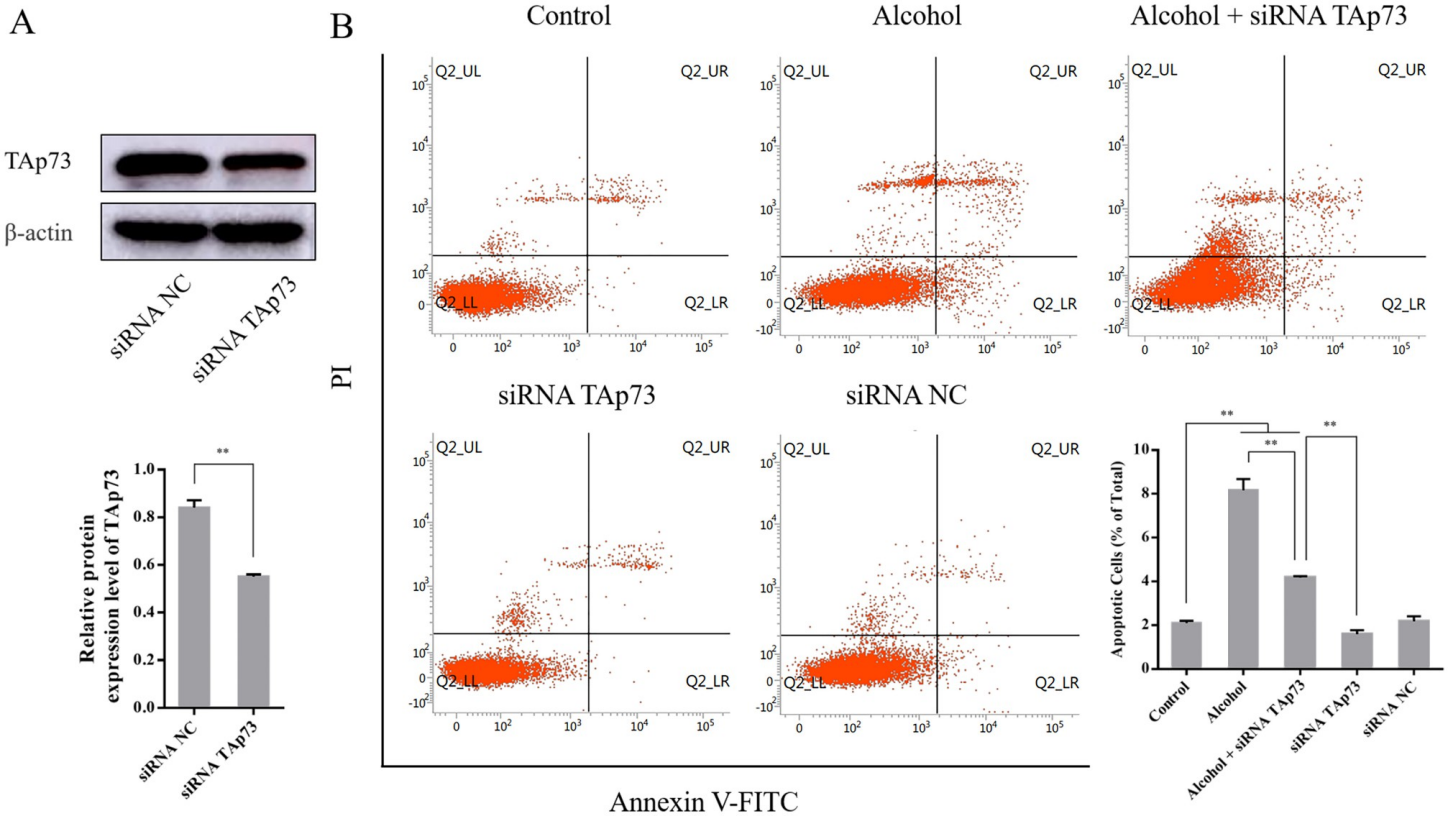
(A) The effect of TAp73 siRNA on the expression of TAp73 in PC12 cells (n = 3). (B) The effect of the siRNA TAp73 on the apoptosis rate of PC12 cells (n = 3). Data information: bars are the mean ± SEM, **P < 0.01 (highly statistically significant).

Compared to the siRNA NC group (0.84 ± 0.03), TAp73 expression was significantly downregulated in the siRNA TAp73 group (0.55 ± 0.01, *P* < 0.01) (Fig 7A).

Compared to the control group (2.11 ± 0.10%), the apoptotic status of PC12 cells stayed at a low level in the siRNA NC group (2.19 ± 0.23%, *P* > 0.05) but were significantly increased in the alcohol group (8.17 ± 0.51, *P* < 0.01) and alcohol + siRNA TAp73 group (4.18 ± 0.06, *P* < 0.01). Compared to the alcohol group, the apoptosis rate of PC12 cells was obviously decreased in the alcohol + siRNA TAp73 group (*P* < 0.01). Finally, compared to the alcohol + siRNA TAp73 group, the apoptosis rate of PC12 cells was significantly decreased in the siRNA TAp73 group (1.60 ± 0.17, P < 0.01) (Fig 7B).

## 4. Discussion

Repeated alcohol exposure can cause permanent neurological damage [12]. The autopsy results of cadavers with long-term drinking histories revealed that 75% of the cases exhibited changes in brain structure and function, accompanied by cognitive dysfunction [13]. Previous research has confirmed that alcohol exposure reduces the volume of brain [14], which may be associated with decreased cell numbers caused by apoptosis [3]. Current research has shown that alcohol can cross the blood–brain barrier and cause oxidative stress in the central nervous system, which can lead to neuronal apoptosis [4]. However, the mechanism of alcohol exposure-induced apoptosis needs to be further explored. The present study will elucidate the detailed mechanism of miR-96-5p and TAp73 involved in alcohol exposure-induced cell injury, which may provide a scientific basis for the prevention and treatment of alcohol-induced damage to the body.

Cell models are efficient ways to identify the underlying mechanism of alcohol-induced neuronal apoptosis. As a cell line derived from rat adrenal pheochromocytoma, differentiated PC12 cells exhibit the general characteristics of neuroendocrine cells and are widely used in neurophysiology and neuropharmacology researches [15, 16]. In this study, after culturing in medium with NGF, differentiated PC12 cells exhibited polygonal or long fusiform morphology with abundant protrusions connected between cells, demonstrating that differentiated PC12 cells have significant structural features of neurons and can be used to probe the in-depth mechanism of neuronal injury.

Current research has shown that alcohol can cross the blood–brain barrier, which can arouse neuronal injury or even death, which can eventually incite a range of neurological dysfunction [4]. In this research, the results of CCK-8 experiments revealed that treatment with alcohol appreciably restrained the activity of PC12 cells, and the results of flow cytometry experiments demonstrated that alcohol significantly increased the apoptosis rate of PC12 cells. The above experimental results indicated that alcohol exposure can induce cell injury or apoptosis, suggesting that PC12 cell model of alcohol-induced neuronal injury has been established successfully.

Several studies have indicated that miR-96-5p personates a vital character in the proliferation and apoptosis of multiple tumor cells and exerts a protective effect against brain damage [17, 18]. Current researches also have indicated that the miR-96-5p can reduce the expression of cysteinyl aspartate specific proteinase 9 (caspase 9), which is an essential promoter in the mitochondrial apoptosis pathway [19]. In this study, the results of flow cytometry experiments illustrated that the miR-96-5p mimic significantly reduces the apoptosis rate of PC12 cells and the miR-96-5p inhibitor significantly increases the apoptosis rate of PC12 cells caused by alcohol exposure, which suggested that miR-96-5p negatively regulates alcohol-induced apoptosis in PC12 cells.

Current examinations have confirmed that miRNAs participates in the adjustment of basic life processes and are related to the occurrence and evolution of multiple diseases [20]. miRNAs are generally believed to inhibit the translation or prompt the degradation of target mRNAs by combining with the untranslated region (3′-UTR) of target genes, regulating gene expression at the transcriptional level [8]. In the present study, the results of dual luciferase report experiment showed that miR-96-5p has a binding target with Tp73. The nucleic acid sequences of miR-96-5p and Tp73 mRNA also indicated that the 3’-UTR regions of Tp73 gene included an overlapping site with the sequence of miR-96-5p. The results of western blot indicated that the mimic for miR-96-5p obviously reduces the expression of TAp73 and that the miR-96-5p inhibitor significantly increases the expression of TAp73. which suggested that miR-96-5p can inhibit the expression of TAP73.

As one of the Tp53 family, Tp73 partakes in the adjustment process of cell cycle arrest and apoptosis [21]. As one of the isomers of Tp73, TAp73 can trigger apoptosis by activating multiple genes, such as Bax, Noxa, and Puma [22]. In the present study, after treating PC12 cells with TAp73 siRNA, the expression of TAp73 and the apoptosis rate of PC12 cells were significantly decreased, suggesting that TAp73 is involved in alcohol-induced apoptosis of PC12 cells.

In a word, this research illustrated that miR-96-5p is involved in alcohol-induced apoptosis of PC12 cells by negatively regulating TAp73. These novel findings provide new evidence for the mechanism of alcohol-induced neuronal injury. However, we need to conduct in-depth follow-up studies in the complex body environment to explore the complete mechanism.

## Supporting information

S1 File (PDF)

S2 File (PDF)

## References

[1] Anderson P. The Impact of Alcoholic Beverages on Human Health. Nutrients. 2021; 13(12): 4417. doi: 10.3390/nu13124417 34959968 PMC8706792

[2] GBD 2016 Alcohol Collaborators. Alcohol use and burden for 195 countries and territories, 1990–2016: a systematic analysis for the Global Burden of Disease Study 2016. Lancet. 2018; 392(10152): 1015–1035. doi: 10.1016/S0140-6736(18)31310-2 30146330 PMC6148333

[3] Mira RG, Lira M, Tapia-Rojas C, Rebolledo DL, Quintanilla RA, Cerpa W. Effect of Alcohol on Hippocampal-Dependent Plasticity and Behavior: Role of Glutamatergic Synaptic Transmission. Front Behav Neurosci. 2020; 13: 288. doi: 10.3389/fnbeh.2019.00288 32038190 PMC6993074

[4] Fadda F, Rossetti ZL. Chronic ethanol consumption: from neuroadaptation to neurodegeneration. Prog Neurobiol. 1998; 56(4): 385–431. doi: 10.1016/s0301-0082(98)00032-x 9775400

[5] Levine AJ, Tomasini R, McKeon FD, Mak TW, Melino G. The p53 family: guardians of maternal reproduction. Nat Rev Mol Cell Biol. 2011; 12(4): 259–265. doi: 10.1038/nrm3086 21427767

[6] Wang J, Xie H, Gao F, Zhao T, Yang H, Kang B. Curcumin induces apoptosis in p53-null Hep3B cells through a TAp73/DNp73-dependent pathway. Tumour Biol. 2016; 37(3): 4203–4212. doi: 10.1007/s13277-015-4029-3 26490992

[7] Logotheti S, Pavlopoulou A, Galtsidis S, Vojtesek B, Zoumpourlis V. Functions, divergence and clinical value of TAp73 isoforms in cancer. Cancer Metastasis Rev. 2013; 32(3–4): 511–534. doi: 10.1007/s10555-013-9424-x 23592418

[8] Morales S, Monzo M, Navarro A. Epigenetic regulation mechanisms of microRNA expression. Biomol Concepts. 2017; 8(5–6): 203–212. doi: 10.1515/bmc-2017-0024 29161231

[9] Nunez YO, Mayfield RD. Understanding Alcoholism Through microRNA Signatures in Brains of Human Alcoholics. Front Genet. 2012; 3: 43. doi: 10.3389/fgene.2012.00043 22514554 PMC3322338

[10] Yi S, Shi W, Zuo M, Wang S, Ma R, Bi H, et al. Endoplasmic Reticulum Stress Is Involved in Glucocorticoid-Induced Apoptosis in PC12 Cells. Anal Cell Pathol (Amst). 2021; 2021: 5565671. doi: 10.1155/2021/5565671 33628710 PMC7895572

[11] Niu S, Shi W, Li Y, Yi S, Li Y, Liu X, et al. Endoplasmic Reticulum Stress Is Associated with the Mesencephalic Dopaminergic Neuron Injury in Stressed Rats. Anal Cell Pathol (Amst). 2021; 2021: 7852710. doi: 10.1155/2021/7852710 34540569 PMC8443372

[12] Kuźma E, Llewellyn DJ, Langa KM, Wallace RB, Lang IA. History of alcohol use disorders and risk of severe cognitive impairment: a 19-year prospective cohort study. Am J Geriatr Psychiatry. 2014; 22(10): 1047–1054. doi: 10.1016/j.jagp.2014.06.001 25091517 PMC4165640

[13] Vetreno RP, Hall JM, Savage LM. Alcohol-related amnesia and dementia: animal models have revealed the contributions of different etiological factors on neuropathology, neurochemical dysfunction and cognitive impairment. Neurobiol Learn Mem. 2011; 96(4): 596–608. doi: 10.1016/j.nlm.2011.01.003 21256970 PMC3086968

[14] Lee J, Im SJ, Lee SG, Stadlin A, Son JW, Shin CJ, et al. Volume of hippocampal subfields in patients with alcohol dependence. Psychiatry Res Neuroimaging. 2016; 258: 16–22. doi: 10.1016/j.pscychresns.2016.10.009 27829188

[15] Pantazis NJ, Dohrman DP, Luo J, Goodlett CR, West JR. Alcohol reduces the number of pheochromocytoma (PC12) cells in culture. Alcohol. 1992; 9(3): 171–180. doi: 10.1016/0741-8329(92)90048-f 1605882

[16] Vincenzi F, Pasquini S, Gessi S, Merighi S, Romagnoli R, Borea PA, et al. The Detrimental Action of Adenosine on Glutamate-Induced Cytotoxicity in PC12 Cells Can Be Shifted towards a Neuroprotective Role through A1AR Positive Allosteric Modulation. Cells. 2020; 9(5): 1242. doi: 10.3390/cells9051242 32443448 PMC7290574

[17] Zhou HY, Wu CQ, Bi EX. MiR-96-5p inhibition induces cell apoptosis in gastric adenocarcinoma. World J Gastroenterol. 2019; 25(47): 6823–6834. doi: 10.3748/wjg.v25.i47.6823 31885423 PMC6931005

[18] Gan J, Cai Q, Qu Y, Zhao F, Wan C, Luo R, et al. miR-96 attenuates status epilepticus-induced brain injury by directly targeting Atg7 and Atg16L1. Sci Rep. 2017; 7(1): 10270. doi: 10.1038/s41598-017-10619-0 28860495 PMC5579030

[19] Iwai N, Yasui K, Tomie A, Gen Y, Terasaki K, Kitaichi T, et al. Oncogenic miR-96-5p inhibits apoptosis by targeting the caspase-9 gene in hepatocellular carcinoma. Int J Oncol. 2018; 53(1): 237–245. doi: 10.3892/ijo.2018.4369 29658604

[20] Kallupi M, Scuppa G, de Guglielmo G, Calò G, Weiss F, Statnick M. A, et al. Genetic Deletion of the Nociceptin/Orphanin FQ Receptor in the Rat Confers Resilience to the Development of Drug Addiction. Neuropsychopharmacology. 2017; 42(3): 695–706. doi: 10.1038/npp.2016.171 27562376 PMC5240182

[21] Nemajerova A, Moll UM. Tissue-specific roles of p73 in development and homeostasis. J Cell Sci. 2019; 132(19): jcs233338. doi: 10.1242/jcs.233338 31582429 PMC6803362

[22] Jancalek R. The role of the TP73 gene and its transcripts in neuro-oncology. Br J Neurosurg. 2014; 28(5): 598–605. doi: 10.3109/02688697.2014.908162 24742294

