# Supplementary material for: miR-96-5p is involved in alcohol-induced apoptosis in PC12 cells via negatively regulating TAp73
Source: PLoS One. 2023 Apr 26;18(4):e0282488. doi: 10.1371/journal.pone.0282488 (PMC10132643; doi:10.1371/journal.pone.0282488)
Supplement: S1 File — (PDF) [file pone.0282488.s001.pdf]

The original blots of Tap73 in Fig.5

The original blots of β-actin in Fig.5

All images were captured by X-ray film with an enhanced chemiluminescence system.

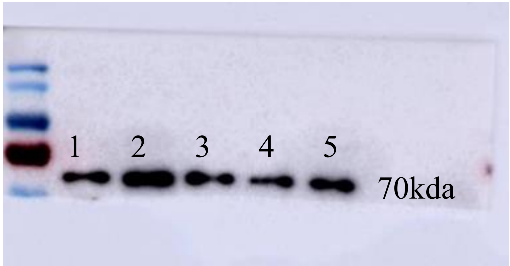

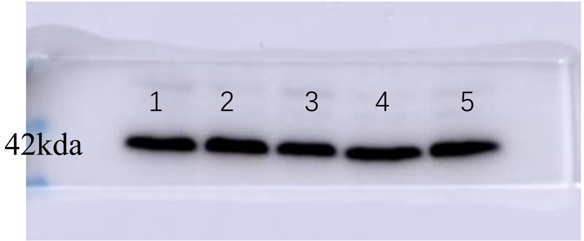

From 1 to 5: Control, Alcohol, Alcohol + mimic, mimic, mimic NC

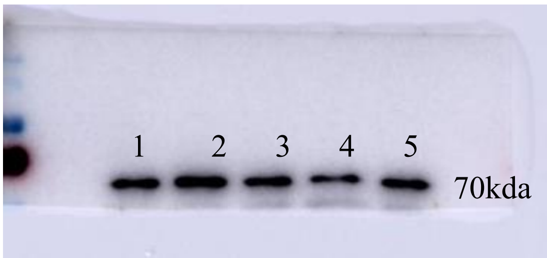

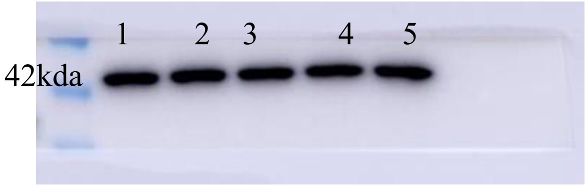

From 1 to 5: Control, Alcohol, Alcohol + mimic, mimic, mimic NC

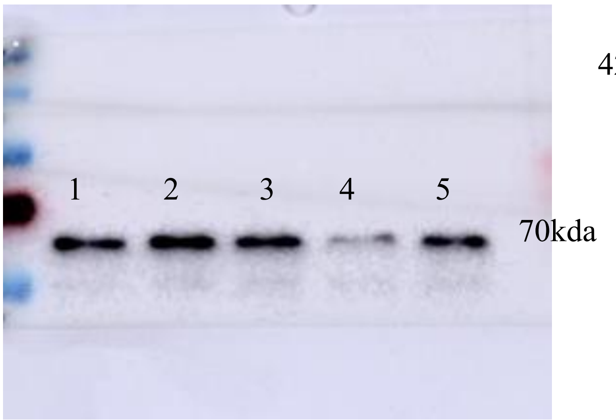

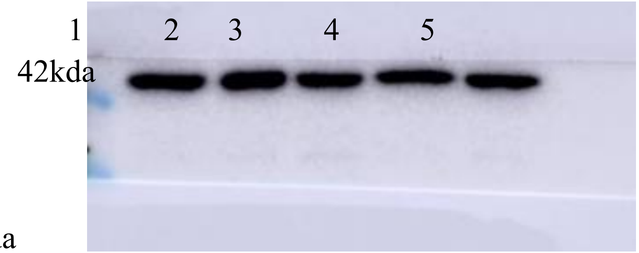

From 1 to 5: Control, Alcohol, Alcohol + mimic, mimic, mimic NC

The original blots of Tap73 in Fig.6

The original blots of β-actin in Fig.6

All images were captured by X-ray film with an enhanced chemiluminescence system.

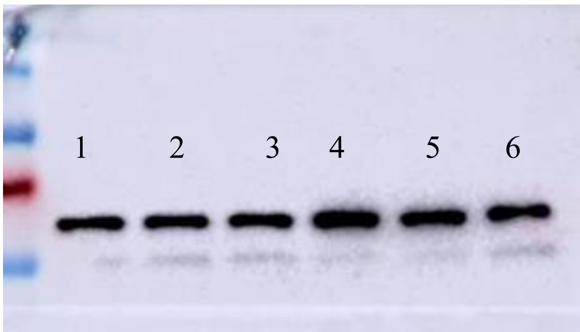

70kda

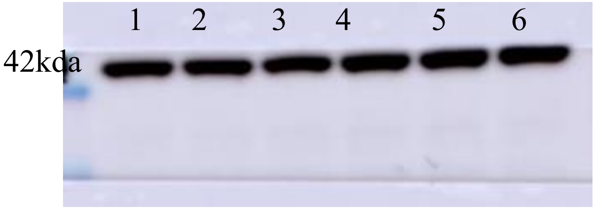

42kda

From 1 to 6: inhibitor NC, inhibitor NC, inhibitor NC, inhibitor, inhibitor, inhibitor

The original blots of Tap73 in Fig.7

The original blots of β-actin in Fig.7

All images were captured by X-ray film with an enhanced chemiluminescence system.

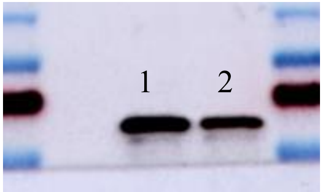

70kda

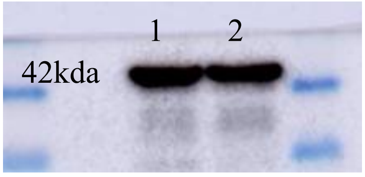

From 1 to 2: siRNA NC, siRNA Tap73

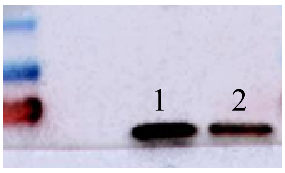

70kda

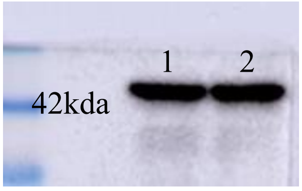

From 1 to 2: siRNA NC, siRNA Tap73

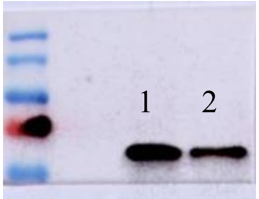

70kda

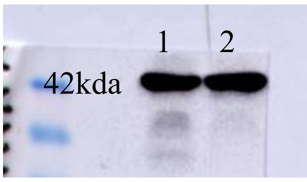

From 1 to 2: siRNA NC, siRNA Tap73
