# Supplementary figures and images for: miR-96-5p is involved in alcohol-induced apoptosis in PC12 cells via negatively regulating TAp73
Source: PLoS One. 2023 Apr 26;18(4):e0282488. doi: 10.1371/journal.pone.0282488 (PMC10132643; doi:10.1371/journal.pone.0282488)

The original pictures of Fig1 B.

Map2

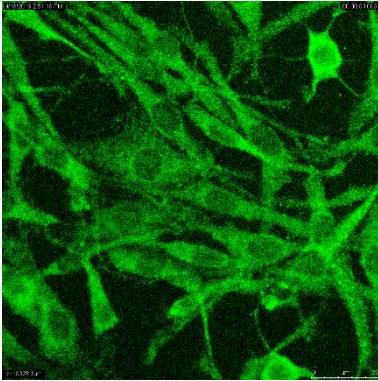

DAPI

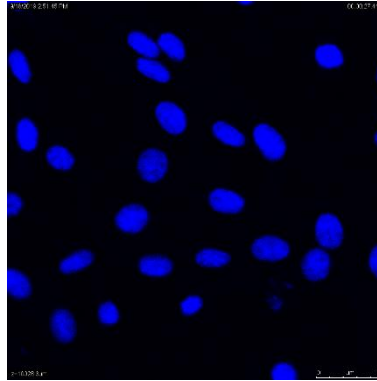

Merge

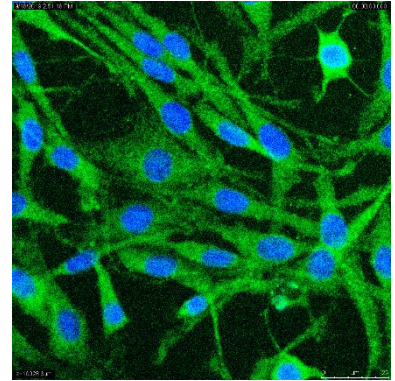

Map2

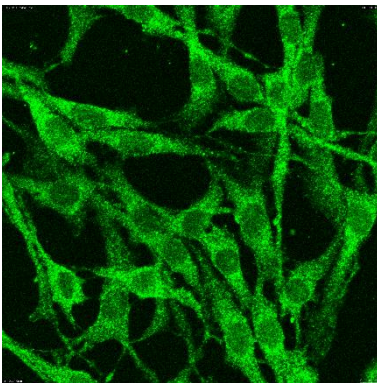

DAPI

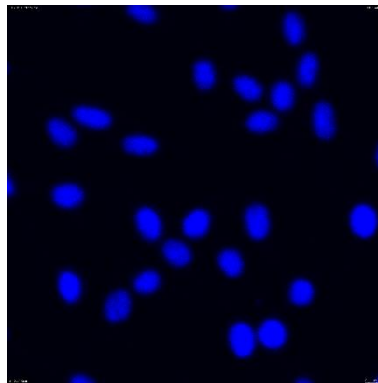

Merge

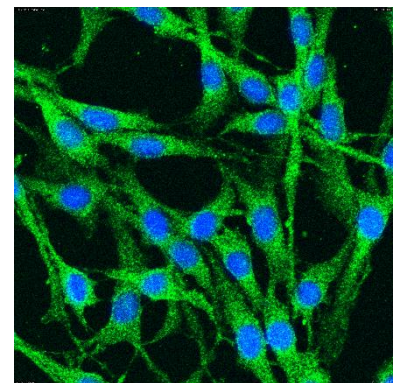

Map2

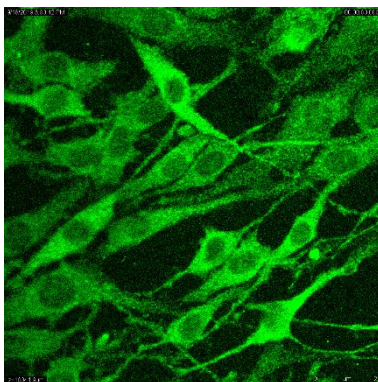

DAPI

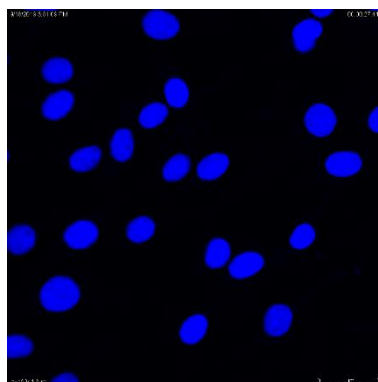

Merge

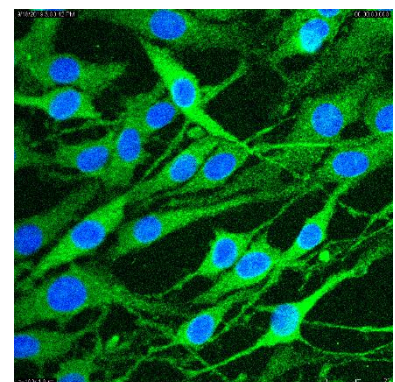

Supplement: S2 File — (PDF) [file pone.0282488.s002.pdf]
